# 

**Published:** 1859-03

**Authors:** 


### LIST OF COLLABORATORS.

S. G. Armor, M.D., late Professor of Pathology and Clinical Medicine, Missouri Med. College.

John L. Atlee, M.D., late President Pennsylvania State Medical Society.

Walter F. Atlee, M.D., Philadelphia.

Robert G. Barclay, M.D., Jerusalem, Syria.

John Bell, M.D., Philadelphia, formerly Professor of Medicine, Medical College of Ohio.

T. S. Bell, M.D., Professor of Medicine, University of Louisville.

S. M. Bemiss, M.D., Professor of Clinical Medicine, University of Louisville.

L. P. Blackburn, M.D., New Orleans, La.

George C. Blackie, M.D., Professor of Botany, University of Nashville.

G. C. Blackman, M.D., Professor of Surgery, Medical College of Ohio.

J. L. Bobbs, M.D., formerly Professor of Anatomy, Indianapolis.

W. M. Boling, M.D., Montgomery, Ala., formerly Professor of Midwifery, Transylvania University, Lexington, Ky.

W. S. Bowen, M.D., New York.

N. Bozeman, M.D., Montgomery, Ala.

J. T. Bradford, M.D., Augusta, Ky.

John H. Brinton, M.D., Lecturer on Operative Surgery, Philadelphia.

W. S. Chipley, M.D., Professor of Medicine, Transylvania University.

A. Clapp, M.D., New Albany, Ia.

D. B. Cliffe, M.D., Franklin, Tenn.

J. Da Costa, M.D., Lecturer on the Practice of Medicine at the Philad. Med. Association.

Samuel H. Dickson, M.D., Professor of Medicine, Jefferson Medical College.

M. Dupierris, M.D., Havana, Cuba.

Richard J. Dunglison, M.D., Philadelphia.

W. F. Edgar, M.D., United States Army.

S. C. Farrar, M.D., Jackson, Miss.

C. S. Fenner, M.D., Memphis, Tenn.

Emil Fischer, M.D., Philadelphia.

Austin Flint, M.D., Professor of Clinical Medicine, Auscultation and Percussion, New Orleans School of Medicine.

Charles Frick, M.D., Baltimore.

W. Y. Gadbury, M.D., Benton, Miss.

O. C. Gibbs, M.D., Chautauque County, New York.

Dr. J. P. HALL, Glasgow, Ky.

WILLIAM A. HAMMOND, M.D., United States Army.

JOHN HARDIN, M.D., Professor of Obstetrics, Kentucky School of Medicine, Louisville.

EDWARD HARTSHORNE, M.D., One of the Surgeons to Wills Hospital for Diseases of the Eye, Philadelphia.

E. B. HASKINS, M.D., Professor of Chemistry, Shelby Medical College, Nashville, Tenn.

C. A. HENTZ, M.D., Marianna, Florida.

ADDINELL HEWSON, M.D., One of the Surgeons to Wills Hospital for Diseases of the Eye, Philadelphia.

SAMUEL L. HOLLINGSWORTH, M.D., late Editor of the Medical Examiner, Philadelphia.

WILSON JEWELL, M.D., Philadelphia, President of the Quarantine and Sanitary Convention.

WM. V. KEATING, M.D., Lecturer on Obstetrics and the Diseases of Women, at the Philadelphia Medical Association.

JOHN G. KERR, M.D., Canton, China.

G. A. KUNKLER, M.D., Madison, Ia.

BEN. LOGAN, M.D., Shreveport, La.

A. LOPEZ, M.D., Mobile, Ala., formerly President of the Alabama State Medical Society.

J. AITKEN MEIGS, M.D., Professor of the Institutes of Medicine in the Philadelphia College of Medicine.

S. WEIR MITCHELL, M.D., Lecturer on Physiology at the Philad. Med. Association.

GEORGE R. MOREHOUSE, M.D., Philadelphia.

B. I. RAPHAEL, M.D., New York.

J. C. REEVE, M.D., Dayton, Ohio.

L. C. RIVES, M.D., formerly Professor of Midwifery, Medical College of Ohio.

J. LAWRENCE SMITH, M.D., Professor of Chemistry, University of Louisville.

W. C. SNEED, M.D., Frankfort, Ky., late President Kentucky State Medical Society.

C. H. SPILMAN, M.D., Harrodsburg, Ky., late President of Kentucky State Medical Society.

GEO. SUTTON, M.D., Aurora, Ia.

W. L. SUTTON, M.D., Georgetown, Ky., formerly President Kentucky State Medical Society.

HORATIO R. STORER, M.D., formerly one of the Physicians to the Boston Lying-in Hospital, Boston, Mass.

S. THOMPSON, M.D., Albion, Ill., late President of the Illinois State Medical Society.

E. WILLIAMS, M.D., Cincinnati, Ohio.

### Bi-Monthly Bibliographical Record.

AMERICAN.

Transactions of the American Medical Association for 1858. Vol. xi. 8vo. pp. 1027. Philadelphia, 1858.

A Treatise on Human Physiology; Designed for the Use of Students and Practitioners of Medicine. By John C. Dalton, M.D., etc. 8vo., pp. 608. Blanchard & Lea: Philadelphia, 1859. (From the author and publishers.)

A Practical Treatise on the Diseases of Children. By D. Francis Condie, M.D., etc. Fifth Edition, revised and enlarged. 8vo., pp. 762. Blanchard & Lea: Philadelphia, 1858. (From the publishers.)

Proceedings of the Academy of Natural Sciences of Philadelphia, as well as of its Biological Department. Philadelphia, 1858.

Physician's Pocket Day-Book, Visiting List, Diary, and Book of Engagements, for 1859. Philadelphia: C. J. Price & Co. (From the publishers.)

Congenital Exstrophy of the Urinary Bladder and its Complications successfully treated by a New Plastic Operation. By Daniel Ayres, M.D., LL.D., etc. Pamphlet, pp. 14. New York, 1859. (From the author.)

Observations on Malarial Fever. By Joseph Jones, A.M., M.D., etc. Articles v., vi., vii. Reprint, 1859. (From the author.)

Contributions to Operative Surgery and Surgical Pathology. By J. M. Carnochan, M.D., etc. Fascic. ii. 4to., pp. 33–77. Philadelphia: Lindsay & Blakiston, 1858. (From the publishers.)

Twentieth Annual Report of the Board of Trustees and Officers of the Central Ohio Lunatic Asylum, for the Year 1858. 8vo., pp. 79. Columbus, 1859.

Report on the Nervous System in Febrile Diseases, and the Classification of Fevers by the Nervous System. By Henry Fraser Campbell, A.M., M.D., etc. Reprint, 8vo., pp. 172. Philadelphia, 1858.

FOREIGN.

The Science and Art of Surgery. By John Erichsen, M.D., etc. An improved American, from the Second enlarged London Edition. 8vo., pp. 996. Philadelphia: Blanchard & Lea, 1859. (From the publishers.)

A Course of Lectures on Obstetrics, delivered at St. Mary's Hospital, London. By Wm. Tyler Smith, M.D., etc. Edited, with Additions, etc., by Augustus K. Gardner, M.D., etc. 8vo., pp. 760. Robert M. De Witt: New York, 1859. (From the editor and publisher.)

The Microscope, in its Application to Practical Medicine. By Lionel Beale, M.B., F.R.S., etc. Second Edition. 8vo., pp. 390. London: John Churchill, 1858. (From the author.)

Locke and Sydenham, with other occasional Papers. By John Brown, M.D., etc. 8vo., pp. 478. Edinburgh: Constable & Co., 1858.

A Syllabus of the Course of Lectures on Medical Logic, delivered in Marischal College and University, Aberdeen. By Francis Ogston, M.D., etc. 12mo., pp. 44. Edinburgh: Maclachan & Stewart, 1858. (From the author.)

Leçons sur le Traitement des Tumeurs Hémorrhoidales par la Méthode de l'Écrasement Linéaire. Par M. E. Chassaignac, M.D., etc. 8vo., pp. 150. Paris: J. B. Ballière et Fils, 1858. (From the publishers.)

A Treatise on the Human Skeleton, including the Joints. By George Murray Humphrey, Esq., F.R.C.S., etc. Cambridge, 1858.

Observations on Diphtheritis. By Willoughby F. Wade, M.B., etc. London: Churchill, 1858. (From the author.)

De la Fièvre Puerpérale; de sa Nature et de sa Traitement. Communications à l'Académie Impériale de Médecine. Par MM. Guérard, Depaul, Beau, Piorry, Hervez de Chégoüi, Trousseau, Paul Dubois, Cruveilhier, Danyau, Cazeaux, Bouillaud, Velpeau, J. Guérin. 8vo. Bossange et Fils: Paris, 1858.

Des Convulsions Urémiques des Femmes Grosses, en Travail et en Couches. Par le Docteur Braun. Traduit par Felix Petard. 8vo. Bossange et Fils: Paris, 1858.

Observations on the Histology, Pathology, and Treatment of Cancerous Diseases. Part i., Melanosis. By Oliver Pemberton, M.D., etc. Royal 8vo. London: Churchill, 1858.

The Urine, in Health and Disease. By Arthur Hill Hassall, M.D. Post 8vo. London: Churchill, 1858.

The Pharmacopœia of the Skin Hospital. By James Startin, M.D., etc. 32mo. London: Churchill, 1858.

On Varicose Veins. By H. T. Chapman, F.R.C.S., etc. Post 8vo. London: Churchill, 1858.

Demonstrations of Diseases in the Chest, and their Physical Diagnosis. By Horace Dobell, M.D., etc. 8vo. London: Churchill, 1858.

Pathologie und Therapie der Muskellähmung. Von Dr. Hermann Friedberg, etc. 8vo., pp. 350. Weimar, 1858.

Morphologie der Menschlichen Nableschnur. Von Dr. Ludwig Adolph Neugebauer. pp. 80. Breslau, 1858.

